# Fixation of unstable sacral fractures by transpedicular system: a prospective study

**DOI:** 10.1007/s00264-025-06673-3

**Published:** 2025-11-14

**Authors:** Mohamed E. Elmoghany, N.O. Gharbo, Mostafa Ahmed Ayoub, Osama Ahmed Farouk, Hosam El-Din Yosry Mashal

**Affiliations:** 1 Tanta University, Tanta, Egypt; 2 Assiut University, Assiut, Egypt

**Keywords:** Unstable sacral fractures, Spinopelvic dissociation, Transpedicular sacral fixation, Lumbopelvic fixation, Sacral decompression

## Abstract

**Purpose:**

This study aimed to assess the functional and radiological outcome of transpedicular fixation system for managing unstable sacral fractures in adults.

**Methods:**

This prospective case series study included 21 patients with unstable type C sacral fractures according to AO Spine classification of sacral fractures. The patients were treated by a transpedicular fixation system connecting the lower lumbar spine to the ilium, as a vertical element, which was bilateral in seven cases and unilateral in 14 cases. A transverse element connecting both sides of the posterior pelvic ring was added to augment fixation in the transverse plane. The minimum period of follow-up was 12 months.

**Results:**

Mean Majeed Score was 84,29 ± 9.97; excellent, good and fair classes were present in 14 (66.7%), five (23.8%) and two (9.5%) patients, respectively. There was a significant reduction of the vertical, anterior posterior and rotational displacement postoperatively in comparison to preoperative measures. There was a significant improvement in neurological deficit postoperatively. Eight (38.1%) patients developed complications postoperatively. Wound Infection was the most common complication.

**Conclusion:**

The use of transpedicular fixation as a vertical element combined with a transverse element connecting both sides of the posterior pelvic ring, to treat unstable sacral fractures, offers adequate fixation strength that helps to achieve union in a well reduced position, leads to satisfactory functional outcome and improves neurological deficit.

**Trial registration:**

(ID/NCT06888583) retrospectively registered.

## Introduction

The sacrum acts as the base of the vertebral column, represents an essential part of the posterior pelvic arch and protects the lumbosacral neurological function [1].

Stable sacral fractures can be treated nonoperatively, while unstable ones usually require surgical intervention. The objectives of surgery include reconstruction of the pelvic ring, restoration of lumbopelvic stability, early mobilization, prevention of fracture displacement, achieving fracture union, and neurological deficit improvement [2–5]. The vertical and transverse axes are the two main biomechanical components that surgeons should consider when deciding on a surgical plan. The fracture type dictates whether it should be fixed in a single plane, or a superadded second plane fixation is needed [6].

Unilateral displaced vertical sacral fractures lateral to L5-S1 facet joint cause disruption of the posterior pelvic ring [7]. Fixation of these fractures in a transverse plane only is usually sufficient in most cases [6].

Conversely, in unilateral sacral fractures, when the fracture line passes through or medial to the L5-S1 facet joint, lumbosacral joint instability is seen [8]. Moreover, spinopelvic dissociation occurs due to sacral fractures that are H-, U-, T-, or lambda-shaped. Fixation in a transverse plane only is not sufficient for these unstable fractures that cause disruption of the spinopelvic junction. These fractures require fixation of the pelvis to the lumbar spine and require fixation in both transverse and vertical planes [9].

There is debate in the literature regarding the technique of reduction, neurological decompression and the ideal fixation method [6, 10, 11].

The objective of this study was to evaluate the radiological and functional results of transpedicular fixation system in the management of unstable sacral fractures in adults.

## Materials and methods

This prospective study was done on 21 patients who presented with AO [12] type C sacral fractures. It was conducted in two level I trauma centres from May 2022 to August 2024 after Ethical Committee approval. All patients signed a written informed consent. The inclusion criteria were closed AO type C sacral fractures in patients who were hemodynamically stable and fit for surgery. The exclusion criteria were patients who were unfit for surgery or bedridden before trauma, and open sacral fractures or with local Morel-Lavallee lesions.

The range of the patients’ age was 18–54 years (mean 26.5+/_9.7). There were 12 males and nine females.

According to AO sacral classification [12], 11 cases were C1, nine cases were C3 and one case was C0.

Eleven cases were operated upon in the first week from trauma, eight cases in the second week, one case in the third week and one case in the sixth week.

### Operative technique

A first-generation cephalosporin was administered intravenous within one hour before skin incision and continued for 24 h postoperatively. On a radiolucent operating table, the patient was positioned in a prone position. The sacrum and lower lumbar spine were exposed through a midline posterior incision. Pedicular screws were inserted in L5 and L4 vertebrae. However, it was difficult to insert L5 transpedicular screws in L5 pedicle in four cases due to severe injury of L5 pedicle, so we extended our fixation proximally to L3 and L4. Iliac pedicle screw entry point was just below the posterior superior iliac spine and directed towards the anterior inferior iliac spine. The correct position of the screw was verified with obturator inlet view and iliac oblique view.

We had seven cases (33.3%) with preoperative neurological affection; two (9.5%) of them underwent direct decompression due to detected fracture fragments in the sacral foramina on CT scan, where sacral laminectomy was performed and the compressing fragments were retrieved from within the sacral canal, while the other five cases (23.8%) underwent indirect decompression by fracture reduction. The hemipelvis was vertically realigned by the assistant’s longitudinal manual traction. A pelvic reduction clamp positioned on the sacral spinous process and iliac crest was used to achieve lateral-to-medial reduction. A ball spike pusher was placed on the lateral surface of the ilium in addition to performing internal rotation of the injured limb to address the external rotation of the hemipelvis. A 7.3 mm partially threaded iliosacral screw (ISS) was used to close the fracture gap and provide adequate compression. If there was sacral dysmorphism, S1 foramen was not visible on C arm, or fracture comminution made it impossible to insert an ISS, we performed either a posterior infix (two iliac pedicular screws connected by a transverse rod), gullwing plate or a transverse connector between the two vertical rods. The transverse element was ISS in nine cases, posterior infix in eight cases, trans-sacral trans-iliac screw in two cases, gullwing plate in one case and a transverse connector between the two vertical rods in one case.

A titanium rod and the necessary clamps were used to connect the pedicle screws of the ilium and lower lumbar spine. The spinopelvic construct was then locked to neutralize the deforming forces. A unilateral spinopelvic construct was done in 14 cases while bilateral construct was done in seven cases. The fixation patterns and constructs used were selected based on the complexity of the fracture. Finally, we put a vacuum drain and closed the wound.

Fourteen (66.6%) cases were associated with anterior pelvic ring injury (six bilateral pubic rami fractures, seven unilateral pubic rami fractures and one symphysis pubis injury). Four cases underwent surgery for fixation of the anterior pelvic ring injury (two cases by anterior plate which was done before spinopelvic fixation (SPF), one case by anterior infix which was done second after SPF and one case by anterior external fixator which was done second after SPF), while the anterior pelvic ring injury was treated conservatively in ten patients. **(**Table 1**)**

**Table 1:** Distribution of the studied cases according to different parameters in total sample (*n* = 21)

	No. (%)
**Sex**	
Male	12(57.1%)
Female	9(42.9%)
**Age**	
< 25	12(57.1%)
≥ 25	9(42.9%)
Min. – Max.	18.0–54.0
Mean ± SD.	26.86 ± 9.77
Median (IQR)	23.0 (20.0–29.0)
**Associated medical comorbidities**	
No	19(90.5%)
Yes	2(9.5%)
**Smoking**	
No	15(71.4%)
Yes	6(28.6%)
**Mechanism of trauma**	
Fall from height	16(76.2%)
Road traffic accident	5(23.8%)
**Fracture classification**	
C0	1(4.8%)
C1	11(52.4%)
C3	9(42.9%)
**Associated anterior pelvic ring injury**	
No injury	7(33.3%)
Unilateral injury	7(33.3%)
Bilateral injury	6(28.6%)
Symphysis pubis disruption	1(4.8%)
**Associated orthopedic injuries**	
No	5(23.8%)
Yes	16(76.2%)
**Associated non-orthopedic injuries**	
No	13(61.9%)
Yes	8(38.1%)
**Time to surgery (weeks)**	
Min. – Max.	1.0–6.0
Mean ± SD.	1.71 ± 1.15
Median (IQR)	1.0 (1.0–2.0)
**Method of fixation**	
Bilateral lumbopelvic fixation	7(33.3%)
Unilateral lumbopelvic fixation	14(66.7%)

Data is presented as mean ± standard deviation & frequency

The mean surgical time was 146.66 ± 20 min, with a range of 90 to 200 min.

The wound was inspected, the drain was removed, and the dressing was changed 48 h postoperatively. Dressing was changed every three days after that, unless there was a concern about infection where daily dressing was done. The patients were advised to avoid lying supine for three weeks after surgery to minimize wound healing problems. Mobilization protocol varied depending on the fracture type and associated injuries. Most patients were allowed to sit and to start passive and active assisted exercises from the second day postoperatively. In unilateral injuries, mobilization using a quad walker with touch down weight bearing was started soon after surgery once the pain was tolerable. In bilateral injuries, it was started six weeks postoperatively with weight bearing mainly on the more stable side.

Functional outcome was assessed by Majeed Score [13] and EQ-5D-5L questionnaire [14] while the radiological outcome was assessed by achievement of fracture union, Henderson [15] and Lefaivre [16] Scoring systems. To evaluate neurological recovery, Gibbons’ grade [17] at the last follow-up was compared to that at presentation.

After surgery, the patients were followed for at least one year.

### Statistical analysis

All collected data was inputted into a computer system and analysed using IBM SPSS 20.0 software. Qualitative data was described using number and percentage, while the Shapiro-Wilk test was utilized to check the normality of distribution. Range (minimum and maximum), mean, standard deviation, median and interquartile range (IQR) were used to describe the quantitative data. The significance between different stages was analysed by the Marginal Homogeneity test. Mann Whitney test was used to compare between two studied groups, for abnormally distributed quantitative variables. Wilcoxon signed ranks test was used for abnormally distributed quantitative variables, to compare between two periods. Spearman coefficient was used to correlate between two abnormally distributed quantitative variables. Significance of the obtained results was judged at the 5% level [18, 19].

## Results

**Fig. 1:**
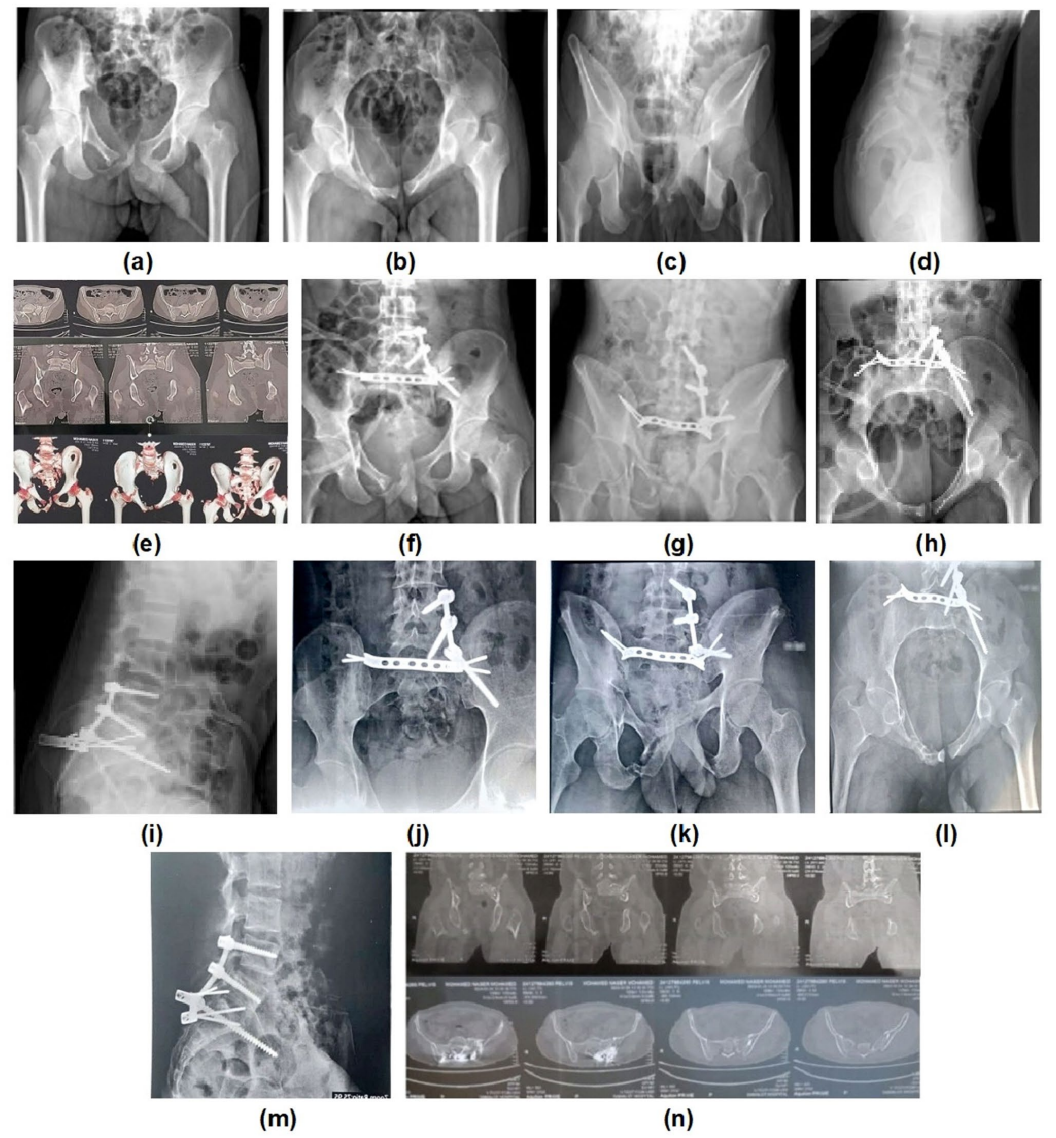
Preoperative (**a, b, c,** and **d**) radiographs of a 22-year-old man, (**e**) Computed tomography showing AO C1 sacral fracture, (**f, g, h** and **i**) Postoperative radiographs, (**j, k, l** and **m**) Follow up radiographs 1 year postoperatively, (**n**) CT showing fracture union

**Fig. 2:**
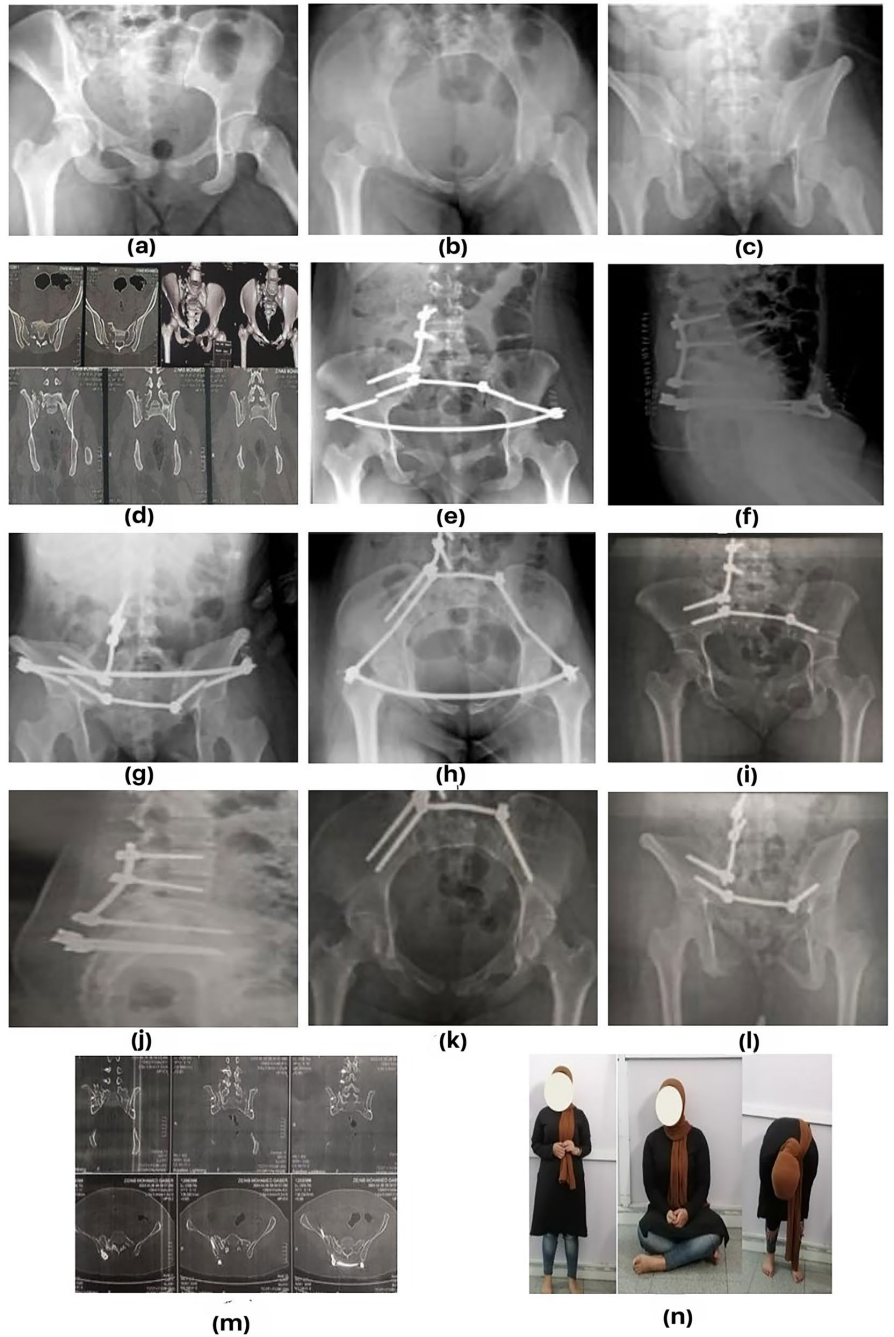
Preoperative (**a, b**, and **c**) radiographs of a 20-year female, (**d**) Computed tomography showing AO C1 sacral fracture, (**e, f, g** and **h**) Postoperative radiographs, (**i, j, k** and **l**) Plain X ray after 1 year showing removal of anterior infix and union of fracture, (**m**) CT showing fracture union, (**n**) Clinical and functional outcome after 12 months

**Fig. 3:**
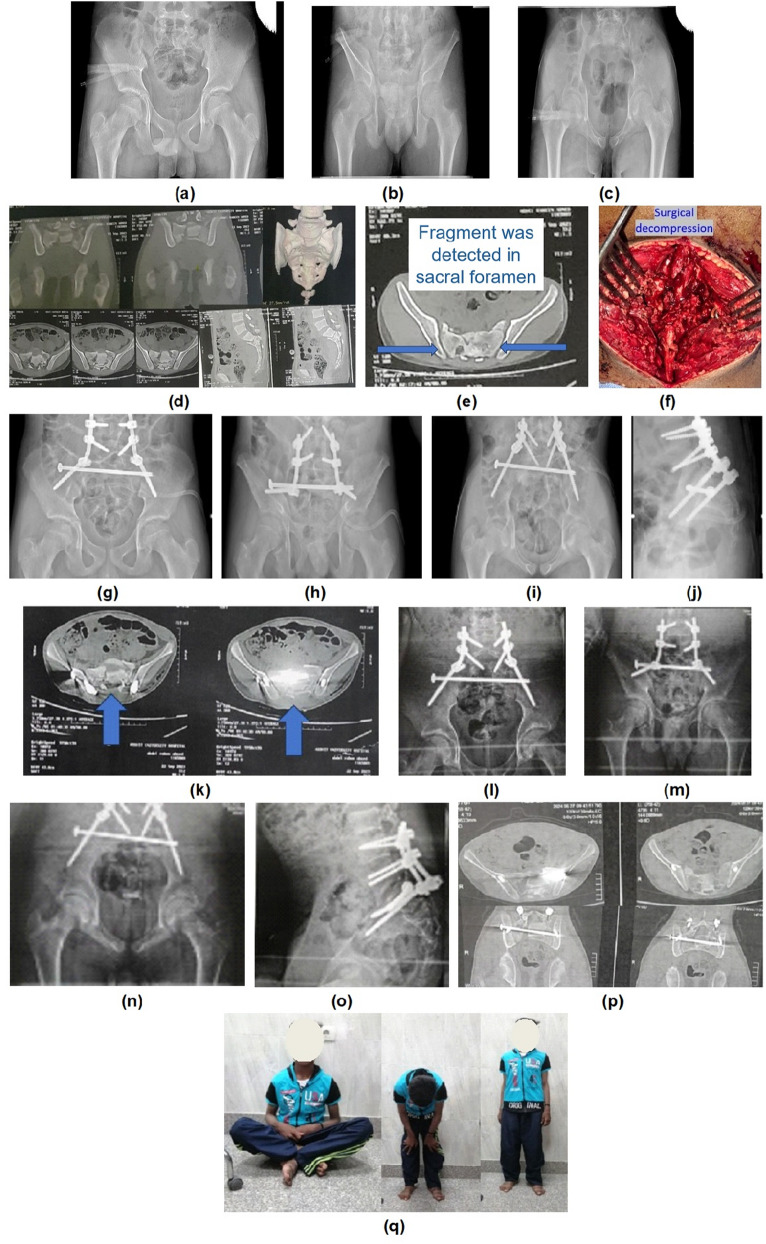
Preoperative (**a, b**, and **c**) radiographs of an 18-year patient, (**d**) Computed tomography showing AO C3 sacral fracture, (**e**) Preoperative CT showing bony fragment in the sacral foramen, (**f**) intraoperative photo showing direct decompression through sacral laminectomy, (**g, h, i** and **j**) Postoperative radiographs, (**k**) Postoperative CT showing sacral decompression, (**l, m, n** and **o**) Follow up radiographs after 1 year, (**p**) CT showing fracture union, (**q**) Clinical and functional outcome after 12 months

Mean Majeed score was 84,29 ± 9.97; excellent, good and fair classes were present in 14 (66.7%), five (23.8%) and two (9.5%) patients, respectively. (Figures 1, 2 and 3)

We used EQ-5D-5L questionnaire as a measure of health status and quality of life; Visual analogue scale mean was 87 ± 10.

**Fig. 4:**
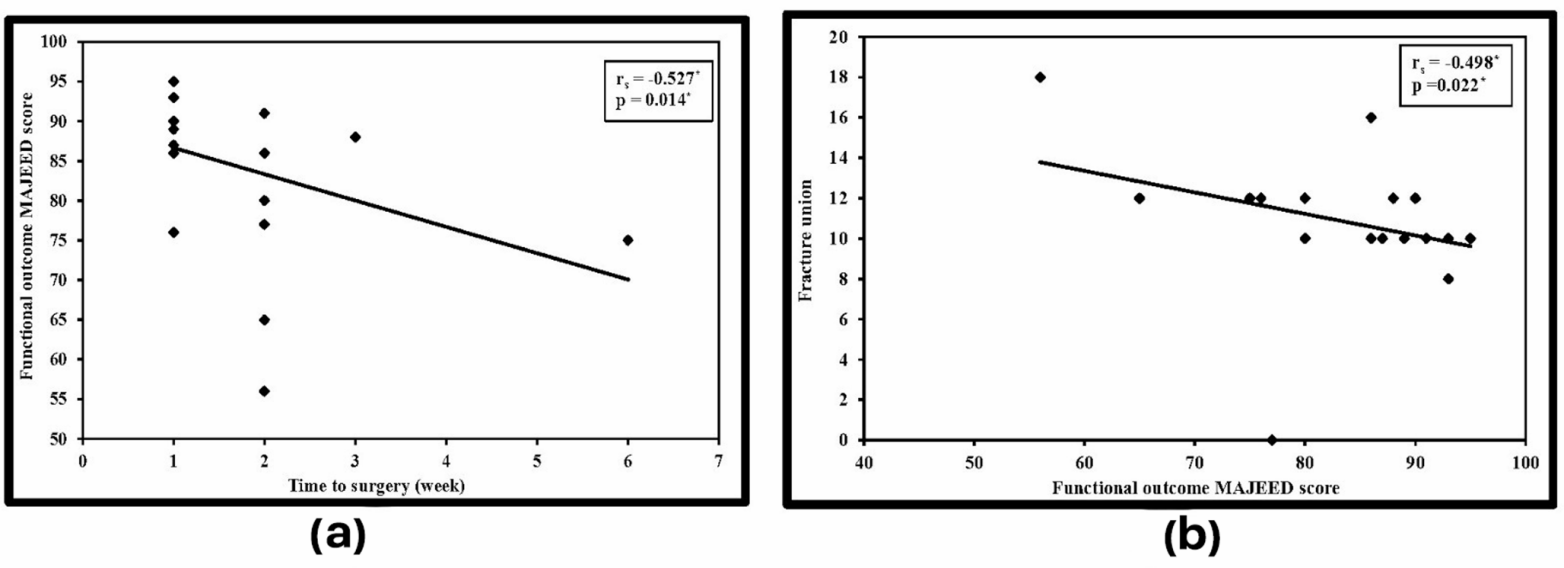
(**a**) Correlation between functional outcome and time to surgery in weeks (*n* = 21), (**b**) Correlation between functional outcome and time to fracture union in weeks (*n* = 21). rs: Spearman coefficient, *: Statistically significant at *p* ≤ 0.05

Functional outcome (Majeed score) was inversely correlated with time lag before surgery, and this was statistically significant. (Fig. 4a)

The time to union ranged between eight weeks to 18 weeks with a mean of 11.3 ± 2.27 weeks. One patient was complicated by non-union because of a loose iliac screw nut with resultant rod dislocation. The patient refused revision surgery and his Majeed score was 77 points. Functional outcome was inversely correlated with time to fracture union, and this was statistically significant. (Fig. 4b)

According to Henderson Scoring system, a significant reduction was observed in the vertical and anteroposterior displacement postoperatively. (Table 2)

**Table 2:** Assessment of reduction of the vertical, anteroposterior and rotational displacements and Gibbons’ grade

	Preoperative	Postoperative	Test of sig.	*p*
**Vertical displacement**				
Fair	2(9.5%)	0(0.0%)	MH = 16.0^*^	0.011^*^
Good	6(28.6%)	2(9.5%)		
Excellent	13(61.9%)	19(90.5%)		
Min. – Max.	0.0–15.0	0.0–6.0	Z = 2.943^*^	0.003^*^
Mean ± SD.	4.29 ± 4.63	1.71 ± 1.71		
Median (IQR)	3.0 (0.0–8.0)	2.0 (0.0–3.0)		
**Anteroposterior displacement**				
Fair	1(4.8%)	0(0.0%)	MH = 12.0^*^	0.034^*^
Good	5(23.8%)	1(4.8%)		
Excellent	15(71.4%)	20(95.2%)		
Min. – Max.	0.0–15.0	0.0–5.0	Z = 2.527^*^	0.012^*^
Mean ± SD.	3.05 ± 4.19	1.29 ± 1.68		
Median (IQR)	0.0 (0.0–7.0)	0.0 (0.0–2.0)		
**Rotational displacement**				
Poor	2(9.5%)	0(0.0%)	MH = 21.50	0.011^*^
Fair	5(23.8%)	2(9.5%)		
Good	8(38.1%)	5(23.8%)		
Excellent	6(28.6%)	14(66.7%)		
Min. – Max.	0.0–25.0	0.0–12.0	Z = 3.629^*^	< 0.001^*^
Mean ± SD.	8.95 ± 7.33	3.52 ± 3.09		
Median (IQR)	7.0 (3.0–13.0)	3.0 (2.0–4.0)		
**Gibbons’ grade**				
Grade 1: No neurological deficit	14(66.7%)	17(81.0%)	MH = 6.50^*^	0.012^*^
Grade 2: Paresthesia only	0(0.0%)	4(19.0%)		
Grade 3: Motor affection	6(28.6%)	0(0.0%)		
Grade 4: Bowel/ bladder affection	1(4.8%)	0(0.0%)		

IQR: Inter quartile range, SD: Standard deviation, MH: Marginal Homogeneity Test, Z: Wilcoxon signed ranks test, p: *p* value for comparing between preoperative and postoperative. *: Statistically significant at *p* ≤ 0.05

According to Lefaivre Scoring system, a significant reduction was observed in the rotational displacement postoperatively. (Table 2)

A statistically significant improvement in Gibbons’ grade was observed postoperatively. (Table 2)

A total of eight (38.1%) patients developed postoperative complications, and some patients had more than one complication. Wound Infection and dehiscence were the most common complications. Five out of 21 patients (23.8%) developed post-operative wound infection. Out of these five patients, one patient had superficial infection that was treated with daily dressing and intravenous antibiotics according to culture and sensitivity, while two patients had deep wound infection that required debridement and closure, another patient had deep infection with wound dehiscence that required debridement with VAC (Vacuum Assisted Closure) then secondary closure, and only one patient had deep infection and wound dehiscence that required multiple sessions of debridement with VAC then a lumbar perforator flap was applied.

Three patients had loose iliac screw nuts; two of them required revision of the loose nut while the third patient refused revision surgery and developed non-union later. One patient had a broken iliac screw after union of the fracture, so we removed all the hardware one year after fixation surgery. One patient developed non-union and one thin patient developed decubitus ulcer over the iliac screw. However, the complications had no significant effect on the final outcome. (Table 3)

**Table 3:** Relation between functional outcome and post-operative complications (*n* = 21)

	*N*	Functional outcome (Majeed score)		U	*p*
		Mean ± SD.	Median (Min. – Max.)		
**Post-operative complications**					
No	**13**	85.92 ± 8.61	89.0 (65.0–93.0)	39.500	0.374
Yes	**8**	81.63 ± 11.99	83.0 (56.0–95.0)		

U: Mann Whitney test, p: *p* value for comparing between different categories

## Discussion

Sacral fractures with spinopelvic instability are challenging injuries. Agreement regarding the technique of reduction, the necessity of decompressing sacral nerve roots, or the most effective fixation technique is yet to be established [6, 10, 11].

Various methods for intraoperative reduction have been documented. Closed reductions, percutaneous methods, and formal open reduction have all been described [20–23]. The technique of reduction we used in our study enabled us to achieve a significant reduction of the vertical, anterior posterior and rotational displacement postoperatively in comparison to preoperative measures.

Regarding fixation techniques, the decision to extend instrumentation to the L4 level and to add a transverse element or not remain a subject of ongoing debate [24–26]. In our study, we preferred to extend instrumentation proximally to include two lumbar vertebrae in addition to adding a transverse element connecting both sides of the posterior pelvic ring, to have a more secure fixation and to guard against secondary displacement.

In our study, the decision to use direct versus indirect neurological decompression was case based. This was typically guided by clinical assessment, the presence of bone fragments inside the canal, the extent of sacral canal narrowing, and intraoperative fracture reducibility assessed by fluoroscopy. Bowel and/or bladder affection was not an absolute indication for direct decompression as long as there was no detected fragment within the canal causing significant compromise. In case of neurological deficit, whether motor or sphincteric, the CT scan was scrutinized. If a significant sacral canal compromise was detected on CT scan, direct decompression through sacral laminectomy was performed. If there was no evident canal compromise, direct decompression was not necessary, but fracture reduction and fixation were required, as soon as possible, to allow recovery of neurapraxia due to the traction injury induced by fracture instability. Accordingly, out of the seven patients who had neurological affection in our study, two underwent direct decompression due to detected fracture fragments in the sacral foramina on CT scan, while the other five cases underwent indirect decompression by fracture reduction. A statistically significant improvement in Gibbons’ grade was observed postoperatively in all our cases. The two patients who underwent direct decompression had preoperative Gibbons’ grade three. Postoperatively, one of them completely recovered, while the other one improved to Gibbons’ grade two. The patient who had preoperative Gibbons’ grade four, underwent indirect decompression through fracture reduction. Postoperatively, she improved to Gibbons’ grade two.

Recent advances in orthopaedic surgery have highlighted the value of preoperative three-dimensional (3D) planning and patient-specific instrumentation in improving surgical precision, especially in anatomically complex regions. Although not yet routinely applied in sacral fracture management, these technologies have shown promising results in various fields. For instance, Russo et al. [27] demonstrated the effectiveness of 3D planning and patient-specific guides in performing corrective osteotomy and shoulder prosthesis implantation in cases of proximal humeral varus malunion. Their approach emphasized the benefits of accurate implant positioning, individualized surgical strategies, and improved anatomical restoration. Additionally, in a systematic review and meta-analysis on 3D printing and fracture mapping in fractures of the acetabulum and pelvis, Lee et al. [28] reported less operative time, blood loss and intraoperative radiation exposure with the use of 3D printing. Moreover, 3D printing resulted in less complications and better reduction. These principles could potentially enhance outcomes if applied in complex spinopelvic reconstructions as well.

SPF was reported to be done through fluoroscopic guided percutaneous techniques [22, 29], and more recently, through minimally invasive robot assisted surgery (MIRAS) [30–32]. Liu et al. [30] used MIRAS in a series of 12 patients and reported a mean operative time of 148.3 ± 40.5 min which is comparable to our study, while Hardigan et al. [31] used MIRAS in a series of seven patients and reported a mean operative time of 178.4 ± 63.9 min which is significantly longer than our study. The mean Majeed score in the study of Liu et al. was 87.2 ± 4.0 which is comparable to our results. Liu et al. had no cases of infection nor non-union, but 2 patients (16.67%) complained of pain due to prominent iliac screw heads. On the other hand, Hardigan et al. reported one iliac screw malposition (14.29%) and one rod dislodgement (14.29%). The complication rate with MIRAS is much less than our study. However, the complications we encountered were manageable and had no significant effect on the final outcome. MIRAS also has the advantages of less blood loss, faster recovery with less pain and better scar cosmesis. However, limitations for MIRAS include higher cost, unavailability in many centers, steep learning curve, and unsuitability for severely displaced fractures where open reduction is required or if direct neurological decompression is needed where a separate incision is necessary for sacral laminectomy [32].

Limitations of our study include the relatively small number of patients, absence of a control group, use of different implants for anterior and posterior fixation, and a relatively short follow-up time.

## Conclusions

The use of transpedicular fixation as a vertical element combined with a transverse element connecting both sides of the posterior pelvic ring, to treat unstable fractures of the sacrum, offers adequate fixation strength that helps to achieve union in a well reduced position, leads to satisfactory functional outcome and improves neurological deficit.

## Data Availability

Data is available on reasonable requests from the corresponding author.
